# Evaluation of Psychiatric Comorbidities and Quality of Life As Well As Brain‐Derived Neurotrophic Factor (BDNF) Concentrations Among Patients Suffering From Acne Vulgaris: A Systematic Review and Meta-Analysis

**DOI:** 10.7759/cureus.33357

**Published:** 2023-01-04

**Authors:** Yaser Mansoor Almutawa, Emma Bhattarai, Muneera AlGhareeb, Jingjun Zhao

**Affiliations:** 1 Dermatology, Department of Dermatology and Venereology, Tongji Hospital, School of Medicine, Tongji University, Shanghai, CHN; 2 College of Medicine and Medical Science, Arabian Gulf University, Manama, BHR

**Keywords:** acne vulgaris, quality of life, depression, anxiety, brain-derived neurotrophic factor (bdnf)

## Abstract

Acne vulgaris usually affects the dermal layer of the skin and is revealed frequently in young adulthood and adolescence. It has serious psychosocial comorbidities. We conducted the present systematic review and meta-analysis to elucidate the association of acne vulgaris with psychiatric comorbidities and quality of life as well as the brain‐derived neurotrophic factor (BDNF) level. A systematic review and meta-analysis of the published articles were carried out following the recommendations of the Preferred Reporting Items for Systematic Reviews and Meta-Analysis (PRISMA) guidelines. We investigated diverse databases: Web of Science, PubMed, the Cochrane Library, Embase, PsycINFO, and CINAHL to search for articles reporting the prevalence of psychosocial comorbidities among patients with acne vulgaris from database inception through June 2022. The outcomes were depression, anxiety, symptom checklist-90-R (SCL-90-R), quality of life, self-esteem, stress, loneliness, and BDNF concentrations. Of 3647 articles identified, 23 met the inclusion criteria. Patients with acne vulgaris have a significantly higher level of anxiety, depression, and stress (P<0.05). Yet, the reported findings of the SCL-90-R, self-esteem, loneliness, and BDNF scores among patients suffering from acne vulgaris were variable and did not differ significantly compared to healthy participants (P>0.05), hampering any conclusive findings on absolute prevalence. Subgroup analysis and comparison showed that heterogeneity between studies was likely due to factors, including country, study design, and assessment tools. This comprehensive review and meta-analysis revealed that anxiety, depression, and stress are significantly more frequent among patients suffering from acne vulgaris. These findings confirm that acne vulgaris has both psychiatric and medical characteristics and requires a multidisciplinary approach.

## Introduction and background

Acne vulgaris, also known as adolescent acne, is characterized by dermal lesions and is the most commonly interpreted and discussed. It is an inflammatory condition affecting the pilosebaceous unit (PSU) [1]. Acne vulgaris is found in zones where an intensive presence of sebaceous glands is detected such as the face. Comedones, which are tiny papules centered by closed comedones (a white spot) or opened comedones (a black spot), are the common basic wounds of acne vulgaris [2].

The Institute for Health Metrics and Evaluation (Global Burden of Disease) reported that this dermis disease occurs in about 9% of the global population with a high incidence among adolescents (85% of people aged between 12 and 25 years) [3]. It ranks among the most common skin disease worldwide, as found in the USA, UK, and France [4]. Four mechanisms have a potent effect on the pathogenesis of acne vulgaris: alteration of the keratinization event inducing comedones; inflammatory mediators released into the PSU; follicular colonization by Propionibacterium acnes; and increased sebum secretion due to androgen regulation.

Acne vulgaris is associated with high psychological and psychosocial disorders and may impact diverse fields of life, inducing social troubles and psychological disorders [5,6]. Acne vulgaris in late adolescence is linked to diverse psychiatric comorbidities, with women being more prone to behavioral and emotional difficulties than men [7]. Many studies have cited problems at school and the absence of love relationships and friendships among patients suffering from acne vulgaris. Other studies have shown that acne vulgaris is associated with diverse conditions like insomnia and attention deficit hyperactivity disease [8,9]. Furthermore, many reports have also revealed that lesions of acne vulgaris present harmful effects on people’s psychiatric well-being and quality of life and may be linked to depression, increasing social isolation, loss of self-esteem, anxiety, and suicide [10,11]. Indeed, depression and anxiety presented a high prevalence among patients with acne, with suicidal cases reaching up to 9-15% of US dermatology patients [11]. Some researchers reported that acne vulgaris may exacerbate psychological stress, but these findings are mostly conflicting and the relationships between acne and psychological stress have not been confirmed yet [12,13].

In this context, we carry out a systematic review and meta-analysis study in order to assess psychiatric comorbidities and quality of life, as well as brain-derived neurotrophic factor (BDNF) level, in patients suffering from acne vulgaris. This information may contribute to better understanding and controlling people with acne vulgaris and its psychiatric burden.

## Review

Materials and methods

Study Design and Database Searching

The present systematic review and meta-analysis study was carried out following Preferred Reporting Items for Systematic Reviews and Meta-Analysis (PRISMA) guidelines [14]. The Embase, Web of Science, PubMed, the Cochrane Library, PsycINFO, and CINAHL databases were used to search potentially interesting articles published from database inception until June 2022. A systematic search was conducted involving all pairwise combinations of Acne vulgaris and these items: ‘‘quality of life’’, ‘‘brain-derived neurotrophic factor’’, ‘‘BDNF’’, ‘‘depress*’’, ‘‘anx*’’, ‘‘psychiat*’’, ‘‘psycho*’’, ‘‘phobia’’, ‘‘stress’’, ‘‘suicide’’, ‘‘loneliness’’, ‘‘self-esteem”. An asterisk was used to add other forms of the terms (ie, psycho* includes psychological or psychosocial).

Selection Criteria

Relevant articles were screened by title and abstract after the suppression of duplicates. Studies were eligible for inclusion if they addressed any psychological or psychosocial evaluation associated with acne vulgaris. The remaining studies were then examined in full text to confirm eligibility.

Inclusion criteria for articles were: (1) non-interventional research (eg: cross-sectional, cohort, or case-control) to investigate the incidence of psychiatric disorders rather than a change in these psychological problems in response to interventions; (2) participants ≥ 12 years; (3) publications evaluating any psychological/psychosocial comorbidities described as outcomes; and (4) publications reporting sufficient data to calculate the effect size (means, standard deviations, and P values from between-group analyses). All included articles performed acne vulgaris evaluation by health care workers. Exclusion criteria for studies were: (1) no full text electronically available; (2) publications in a language other than English, (3) letters, editorials, comments, protocols, review papers, and guidelines; and (4) articles with limited outcome information.

Data Extraction

Two independent authors retrieved information from the eligible articles following the inclusion and exclusion criteria, and information was collected on a standardized data sheet that included the author's name, year, type of study, geographic origin, sample size, age of participants, and outcome ascertainment.

Quality Assessment

Newcastle-Ottawa Scale (NOS) was used to assess the quality of the non-randomized studies, which evaluates selection bias, comparability of the exposed and control participants, and outcome evaluation. Each criterion was assessed as 1 star or 0 stars. The total stars of the NOS checklist ranged from 0 to 9. The NOS tool evaluates three sections: (1) selection of exposed (patients) and unexposed groups (control group) (max 4 stars), (2) comparability of study groups (max 2 stars), and (3) evaluation of outcomes/exposure (max 3 stars). Two independent authors assessed quality independently and discordances were solved by discussion. A study with a score from 7 to 9 has high quality, 4 to 6 has moderate quality, and 0 to 3 has low quality [15].

Measures

Here, we have analyzed 12 measures, which were categorized into five groups: quality of life (Acne Quality of Life Scale (AQOL) and Diabetes Quality of Life (DQOL)), SCL-90-R, anxiety outcomes (Hospital Anxiety and Depression Scale-Anxiety subscale (HADS-A), Liebowitz Social Anxiety Scale (LSAS), and Anxiety Sensitivity Index-3 (ASI-3)), depression outcomes (Hospital Anxiety and Depression Scale-Anxiety subscale-Depression subscale (HADS-D) and Beck Depression Inventory (BDI)), diverse psychosocial outcomes (Rosenberg Self-Esteem Scale (RSS), Perceived Stress Scale (PSS), and University of California Los Angeles Loneliness Scale (UCLA-LS)), and BDNF.

Quality of Life

Dermatology Life Quality Index (DLQI) scales described by Finlay and Khan concern patients' perception of the effect of skin conditions on diverse features of their health-related quality of life. It is composed of 10 units and four scores (0, “not at all”; 3, “very much”) [16]. AQOL, developed by Gupta et al., is a health-related quality-of-life measure that contains nine modules [17]. AQOL consists of a four-point rating scale (0: ‘not at all’ and 3: ‘very markedly’). Through this questionnaire, patients evaluate the association between acne seriousness and quality of life, especially those presenting moderate or severe forms of acne vulgaris.

SCL-90-R

SCL-90-R is a multidimensional questionnaire, which is created to detect a scale of psychopathological traits and psychiatric symptoms. It is composed of 90 symptoms and evaluated nine psychological dimensions: obsessive compulsion, somatization, depression, interpersonal sensitivity, hostility, anxiety, psychoticism, phobic anxiety, and paranoid ideation [18].

Anxiety

ADS, described by Zigmond and Snaith in 1983, contains 14 scales, seven related to anxiety (HADS-A) and seven to depression (HADS-D) [19]. HADS is considered a sure, effective, and powerful scale to evaluate anxiety and depression. High scores indicate worse anxiety and depression levels. LSAS, described by Liebowitz et al. in 1987, is a 24-item questionnaire [20]. LSAS is a reliable and valid tool to evaluate how social phobia is involved in life across a wide range of situations [20]. ASI-3 estimates fear of anxiety-related emotions [21]. It assesses the three most cantilevered anxiety sensitivity fields: physical, cognitive, and social.

Depression

BDI was invented by Beck et al. [22]. It is scored from 0 to 3 and contains 21 items.

Diverse Psychosocial Outcomes

Rosenberg Self-Esteem Scale (RSS), developed by Morris Rosenberg, is a self-esteem tool extensively used in social research [23]. RSS is a 4-point Likert scale ranging from strongly agree to strongly disagree. PSS, developed by Cohen et al. in 1983, is a 10-item questionnaire, which is the most frequently used psychiatric tool to measure the perception of stress [24]. University of Carolina Los Angeles loneliness scale (UCLA-LS) was described by Russel et al. [25]. High scores are associated with high levels of loneliness.

BDNF

BDNF belongs to the neurotrophin group, and it contributes to many cellular phenomena like proliferation, survival, and maintenance of neurons. It was shown that BDNF might be implicated in the development of certain skin diseases, which could be worsened by stress like psoriasis and vitiligo [26].

Statistical Analysis

RevMan V5.4 (Cochrane Collaboration, Oxford, United Kingdom) was used to conduct the statistical analysis. Mean difference (MD) with 95% confidence intervals (CIs) was calculated to evaluate all the outcomes. A value of P<0.05 was considered as the level of significance. The Cochrane chi-squared test was conducted to evaluate heterogeneity among articles, with a P-value < 0.05 indicating the existence of heterogeneity. Indeed, Cochran's chi-squared test is the traditional test for heterogeneity in meta-analyses. Based on a chi-square distribution, it generates a probability that, when large, indicates larger variation across studies rather than within subjects within a study. I^2^ describes the percentage of the variability and estimates that is due to heterogeneity rather than sampling error (chance). Thresholds for the interpretation of the I^2^ statistic can be misleading since the importance of inconsistency depends on several factors. A rough guide to interpretation in the context of meta-analyses of randomized trials is as follows: 0% to 40%: might not be important; 30% to 60%: may represent moderate heterogeneity; 50% to 90%: may represent substantial heterogeneity; 75% to 100%: may represent considerable heterogeneity. The importance of the observed value of I^2^ depends on the strength of evidence for heterogeneity (P-value from the chi-square test). Hence, I^2^ values ≥ 50% and P < 0.05 indicated a moderate to a high degree of heterogeneity among pooled studies while I^2^ values < 50% and P > 0.05 indicated a low degree of heterogeneity. A fixed-effects design was used when I^2^ < 50% and P > 0.05; otherwise, a random-effects model was adopted [27]. We also performed subgroup and sensitivity analyses to assess the possible source of heterogeneity. Egger's test is commonly used to assess potential publication bias in a meta-analysis via funnel plot asymmetry (Egger's test is a linear regression of the intervention effect estimates on their standard errors weighted by their inverse variance). A value of P <0.05 indicated the presence of publication bias. This test was conducted via Statistical Package for Social Sciences (SPSS) version 25 (IBM Corp., Armonk, NY). Publication bias was further assessed based on the visual inspection of the symmetry in funnel plots.

Results

Studies Identification

**Figure 1:**
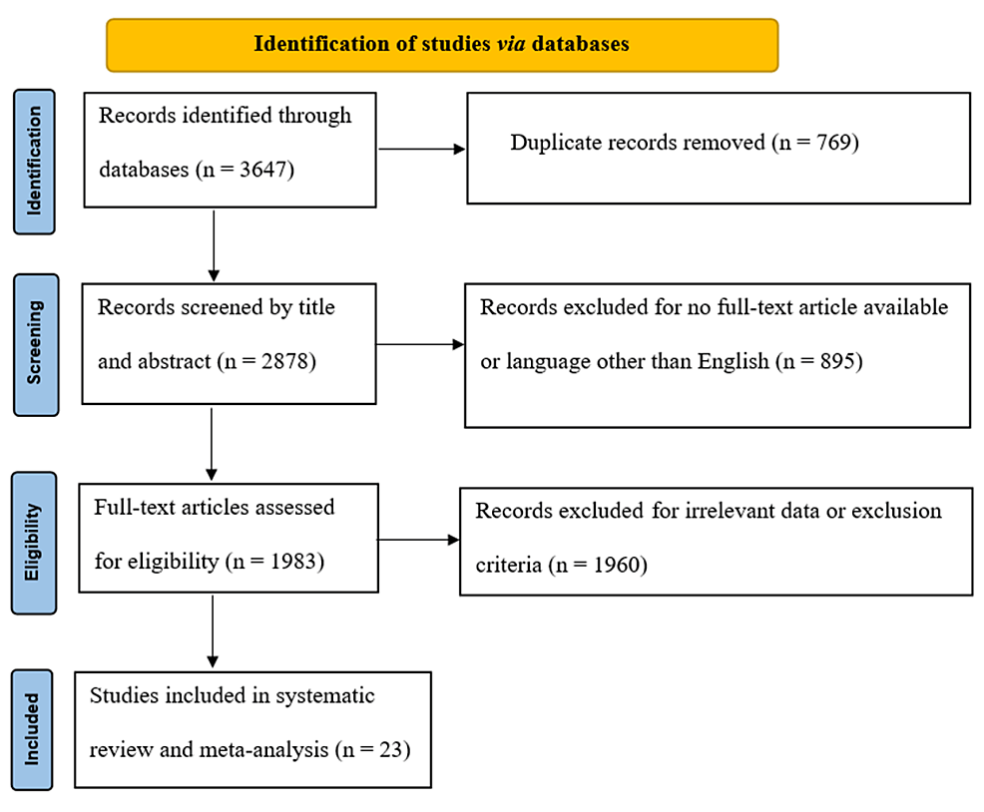
PRISMA flow diagram outlining the selection of studies for this systematic review and meta-analysis PRISMA: Preferred Reporting Items for Systematic Review and Meta-Analyses

Database searching identified 3647 studies to be screened, of which 1983 abstracts were revealed as potentially eligible and retrieved for full-text review. Eligibility criteria were met by 23 articles, which belonged to this systematic review and meta-analysis study. The PRISMA study flowchart is presented in Figure 1.

Features of Included Articles

All the included articles were issued between 2000 and 2021 and were distributed among five countries. The majority of studies were conducted in Turkey (15/23, 65.22%). Among the 23 articles that belonged to this systematic review and meta-analysis, 14 were case-control studies and eight used a cross-sectional study design. Twelve measures, which were the most investigated in the 23 articles, were used in this systematic review and meta-analysis: Beck Depression Inventory (BDI), Hospital Anxiety (HADS-A) and Depression (HADS-D) scales, Rosenberg Self-Esteem Scale (RSS), Symptom Check List-90-Revised (SCL-90-R), Anxiety Sensitivity Index (ASI-3), Brain‐Derived Neurotrophic Factor (BDNF), Perceived Stress Scale (PSS), University of California Los Angeles Loneliness Scale (UCLA-LS), and Liebowitz Social Anxiety Scale (LSAS). The sample size of the included articles varied from 56 to 2284. In summary, the total number of participants was 7809 with 4689 patients with acne vulgaris and 3120 healthy participants. The studies’ characteristics are summarized in Table 1.

**Table 1:** List of the articles included in the meta-analysis with their year of publication, geographic distribution, type of study, participant characteristics, and outcome ascertainment RSS: Rosenberg Self-Esteem Scale; BDI: Beck Depression Inventory; STAI: State-Trait Anxiety Inventory; AQOL: Acne Quality of Life Scale; STAXI: Spielberger State-Trait Anger Expression Inventory; DLQI: Dermatology Life Quality Index; HADS: Hospital Anxiety and Depression Scale; SCL-90-R: Symptom Check List-90-Revised; CFSEI-AD: Culture-Free Self-Esteem Inventory-Adult Version; LSAS: Liebowitz Social Anxiety Scale; MOCQ: Maudsley Obsessive Compulsive Questionnaire; SDS: Sheehan Disability Scale; AIS Athens Insomnia Scale; SF-36: Short Form-36 (health-related quality of life scale); TCI: Temperament and Character Inventory; EPQ-RSF: Eysenck Personality Questionnaire-Revised Short Form; ASLEC: Adolescent Self-Rating Life Events Check; CSPSCA: Capa Social Phobia Scale for Children and Adolescents; ASI-3: Anxiety Sensitivity Index; BDNF: Brain‐Derived Neurotrophic Factor; PSS: Perceived Stress Scale; SPS: Suicide Probability Scale; LSS: Life Satisfaction Scale; MDA, Malondialdehyde; TAC, Total Antioxidant Capacity; DERS-16: Difficulties in Emotion Regulation Scale; PHQ-9: 9-Item Patient Health Questionnaire; YIAS-SF: Young Internet Addiction Scale-Short Form, BAI: Beck Anxiety Index; UCLA-LS: University of California Los Angeles Loneliness Scale; SAAS: Social Appearance Anxiety Scale

			Participants				
Article, year	Country	Study design	N	Acne Vulgaris (N)	Control group (N)	Age	Outcome
Sayar et al., 2000 [28]	Turkey	Case-control study	56	31	25	13-30	BDI, RSS, STAI, STAXI
Aktan et al., 2000 [29]	Turkey	Case-control study	616	308	308	14-20	HADS
Yazici et al., 2004 [30]	Turkey	Case-control study	99	61	38	16-26	AQOL, DLQI, HADS
Abdel-Hafez et al., 2009 [31]	Egypt	Case-control study	200	150	50	17-25	DLQI, SCL-90-R, CFSEI-AD
Golchai et al., 2010 [32]	Iran	Cross-sectional study	164	82	82	14-30	HADS
Bez et al., 2011 [33]	Turkey	Case-control study	238	140	98	15-33	LSAS, HADS, SDS
Bez et al., 2013 [34]	Turkey	Case-control study	240	146	94	15-38	HADS, SF-36, MOCQ
Öztürk et al., 2013 [35]	Turkey	Case-control study	87	47	40	16-47	TCI, HADS
Wen et al., 2015 [36]	China	Case-control study	2284	1,156	1,128	15-25	ASLEC, HADS
Gül & Çölgeçen., 2015 [37]	Turkey	Case-control study	80	40	40	19-55	SCL 90-R, EPQ-RSF
Unal et al., 2018 [38]	Turkey	Case-control study	183	102	81	12-17	CSPSCA, RSS, AQOL
Duman et al.,2016 [39]	Turkey	Case-control study	225	125	100	14-35	HADS
Salman et al., 2016 [40]	Turkey	Cross-sectional study	74	37	37	20-24	LSAS, HADS, DLQI
Sereflican et al., 2019 [41]	Turkey	Cross-sectional study	122	61	61	>16	LSAS, HADS, SDS, ASI-3, PSS
Mikhael et al., 2019 [26]	Egypt	Case-control study	80	60	20	18-22	BDNF, PSS, DLQI, HADS
Özyay Eroğlu et al., 2019 [42]	Turkey	Cross-sectional study	206	104	102	14-18	SPS, RSS, LSS, UCLA-LS
Awad et al., 2018 [43]	Egypt	Case-control study	100	60	40	17-30	HADS, MDA, TAC, Zinc
Acer et al., 2019 [44]	Turkey	Cross-sectional study	331	214	117	18-24	ASI-3, BAI
Cengiz & Gürel, 2020 [45]	Turkey	Cross-sectional study	243	141	102	18-37	DERS, HADS, AQOL
He et al., 2019 [46]	China	Cross-sectional study	177	118	59	19-21	BDNF, IL-6, TNF-α, PHQ-9, AIS
Öztekin & Öztekin, 2020 [47]	Turkey	Cross-sectional study	405	203	202	19-25	YIAS-SF, AQOL, UCLA-LS, BDI
Duru et Orsal, 2021 [48]	Turkey	Cross-sectional study	1007	1007	0	17-36	AQOL, SAAS
Molla et al., 2021 [49]	Saudi Arabia	Case-Control Study	592	296	296	12-60	HADS

*Quality Assessment* 

Overall, the scores of included studies ranged from five to eight stars. Among the included studies, 15 were assessed to be of high quality while 8 were of moderate quality. Table 2 summarized the quality assessment scores for the included studies. The majority of the selected studies (15/23) scored 3 stars, while 6 scored 2 stars and only two studies received a full quality score for the selection section (4 stars). The reasons for not receiving a full quality score for the selection section were that (1) the controls were recruited in a hospital setting, or recruiting was not explained at all, (2) the sample size was not justified, and (3) no description of the response rate or the characteristics of the responders and non-responders. Among the included studies, 19 studies controlled for the outcomes and additional factors (e.g., age) and scored two stars. However, three studies controlled for only the outcomes and scored one star while only one study did not include a control group. All the case-control studies reported the ascertainment of the outcome and used the same method of ascertainment for cases and controls, so they scored two stars. All cross-sectional studies adopted a validated assessment tool and used an adequate and appropriate statistical analysis; thus, they scored two stars.

**Table 2:** Modified Newcastle-Ottawa quality assessment scale for the included studies

Study	Selection	Comparability	Outcome	Quality score
Sayar et al., 2000 [28]	★★★	★★	★★	7 (High quality)
Aktan et al., 2000 [29]	★★	★	★★	5 (Moderate quality)
Yazici et al., 2004 [30]	★★★	★★	★★	7 (High quality)
Abdel-Hafez et al., 2009 [31]	★★	★★	★★	6 (Moderate quality)
Golchai et al., 2010 [32]	★★★	★★	★★	7 (High quality)
Bez et al., 2011 [33]	★★★★	★★	★★	8 (High quality)
Bez et al., 2013 [34]	★★★★	★★	★★	8 (High quality)
Öztürk et al., 2013 [35]	★★	★★	★★	6 (Moderate quality)
Wen et al., 2015 [36]	★★	★	★★	5 (Moderate quality)
Gül & Çölgeçen., 2015 [37]	★★★	★★	★★	7 (High quality)
Unal et al., 2018 [38]	★★	★★	★★	6 (Moderate quality)
Duman et al.,2016 [39]	★★★	★★	★★	7 (High quality)
Salman et al., 2016 [40]	★★★	★★	★★	7 (High quality)
Sereflican et al., 2019 [41]	★★★	★★	★★	7 (High quality)
Mikhael et al., 2019 [26]	★★★	★	★★	6 (Moderate quality)
Özyay Eroğlu et al., 2019 [42]	★★★	★★	★★	7 (High quality)
Awad et al., 2018 [43]	★★★	★★	★★	7 (High quality)
Acer et al., 2019 [44]	★★★	★★	★★	7 (High quality)
Cengiz & Gürel, 2020 [45]	★★★	★★	★★	7 (High quality)
He et al., 2019 [46]	★★★	★★	★★	7 (High quality)
Öztekin & Öztekin, 2020 [47]	★★	★★	★★	6 (Moderate quality)
Duru et Orsal, 2021 [48]	★★★		★★	5 (Moderate quality)
Molla et al., 2021 [49]	★★★	★★	★★	7 (High quality)

Outcome Measures

**Figure 2:**
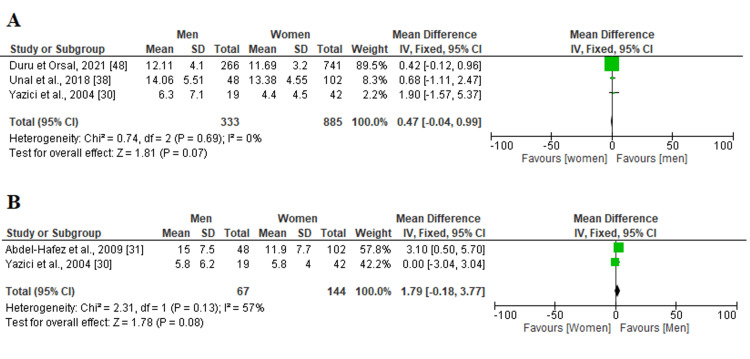
Forest plot representing the mean difference (MD) of (A) AQOL and (B) DQOL scores between men and women in the patient group SD: standard deviation, IV: inverse variance, CI: confidence interval; AQOL: Acne Quality of Life Scale; DQOL: Diabetes Quality of Life The solid vertical line represents a mean difference of 0 or no effect. Each square around the point effect represents the mean effect size for that study and reflects the relative weighting of the study to the overall effect size estimate. The larger the box, the greater the study's contribution to the overall estimate. The upper and lower limit of the line connected to the square represents the upper and lower 95% CI for the effect size.

Quality of life: Two measures analyzing the quality of life belonged to this systematic review and meta-analysis (AQOL and DQOL). Among the 23 included studies, AQOL and DQOL were evaluated by three and two studies, respectively. The heterogeneity (P > 0.05, I^2 ^< 75%) was low in both scales, so a fixed effect model was used (Figure 2). We noticed that no notable difference was detected among genders in patients suffering from acne vulgaris in terms of AQOL and DQOL measures (P > 0.05).

**Figure 3:**
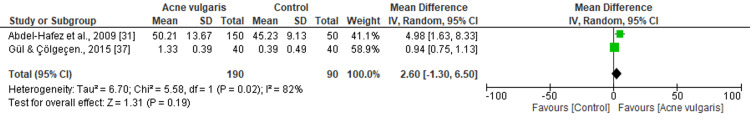
Forest plot of the estimated mean difference of SCL-90-R subscale scores between patients and the control group SD: standard deviation; IV: inverse variance; CI: confidence interval; SCL-90-R: Symptom Check List-90-Revised The solid vertical line represents a mean difference of 0 or no effect. Each square around the point effect represents the mean effect size for that study and reflects the relative weighting of the study to the overall effect size estimate. The larger the box, the greater the study's contribution to the overall estimate. The upper and lower limit of the line connected to the square represents the upper and lower 95% CI for the effect size.

SCL-90-R:* *Among 23 included studies, two reported SCL-90-R, with a total of 280 participants. The heterogeneity (chi^2^=5.58, P=0.02, I^2^=82%) was high, so we used a random-effects design (Figure 3). We noticed that the difference in terms of SCL-90-R subscale scores between patients and controls was not statistically significant (MD=2.60; 95% CI: -130 to 6.50; P=0.19).

**Figure 4:**
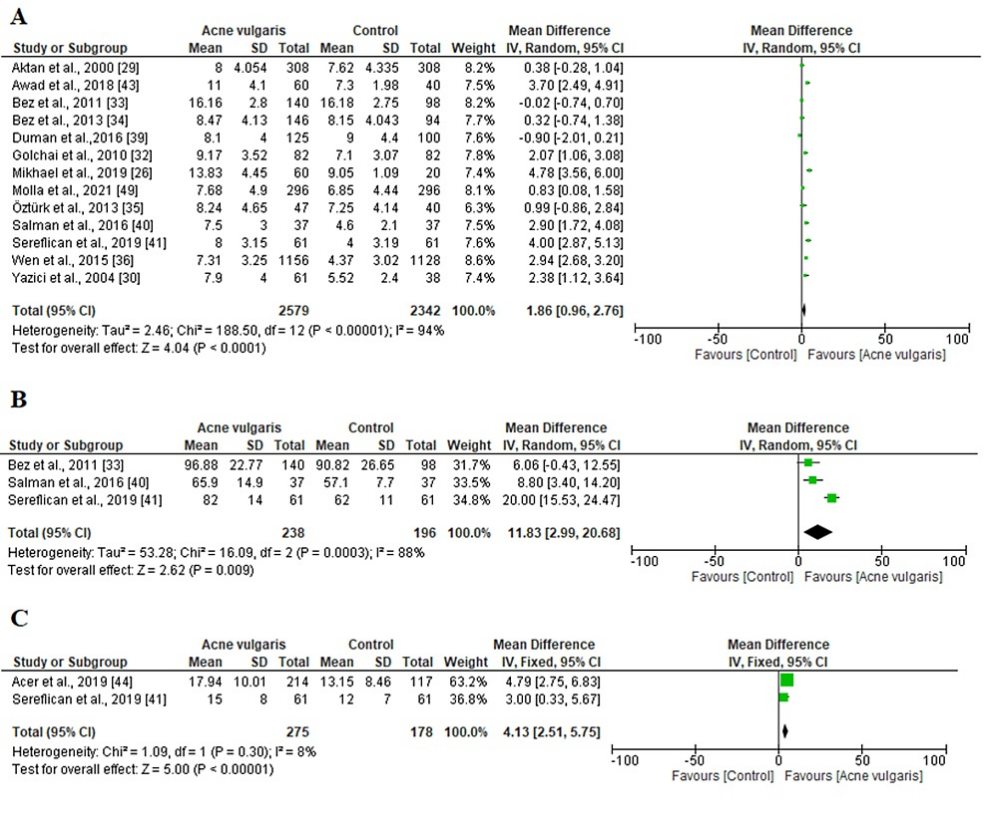
Forest plots representing the estimated mean difference (MD) of (A) HADS-A, (B) LSAS, (C) ASI-3 scores between patients and the control group SD: standard deviation; IV: inverse variance; CI: confidence interval; HADS-A: Hospital Anxiety and Depression Scale-Anxiety Subscale; LSAS: Liebowitz Social Anxiety Scale; ASI-3: Anxiety Sensitivity Index-3 The solid vertical line represents a mean difference of 0 or no effect. Each square around the point effect represents the mean effect size for that study and reflects the relative weighting of the study to the overall effect size estimate. The larger the box, the greater the study's contribution to the overall estimate. The upper and lower limits of the line connected to the square represent the upper and lower 95% CI for the effect size.

Anxiety outcomes: Three outcomes analyzing anxiety were investigated in this systematic review and meta-analysis (HADS-A, LSAS, and ASI-3). Among 23 included studies, eight, three, and two studies reported the HADS-A, LSAS, and ASI-3 outcomes, respectively. A random-effects design was adopted for HADS-A and LSAS outcomes because the heterogeneity was high (P < 0.05, I^2^ > 80%). However, we used a fixed-effects design for the ASI-3 outcome considering that the heterogeneity was low (chi^2^=1.09, P=0.30, I^2^=8%) (Figure 4). The three forest plots showed that anxiety was significantly higher among patients with acne vulgaris (P < 0.05).

**Figure 5:**
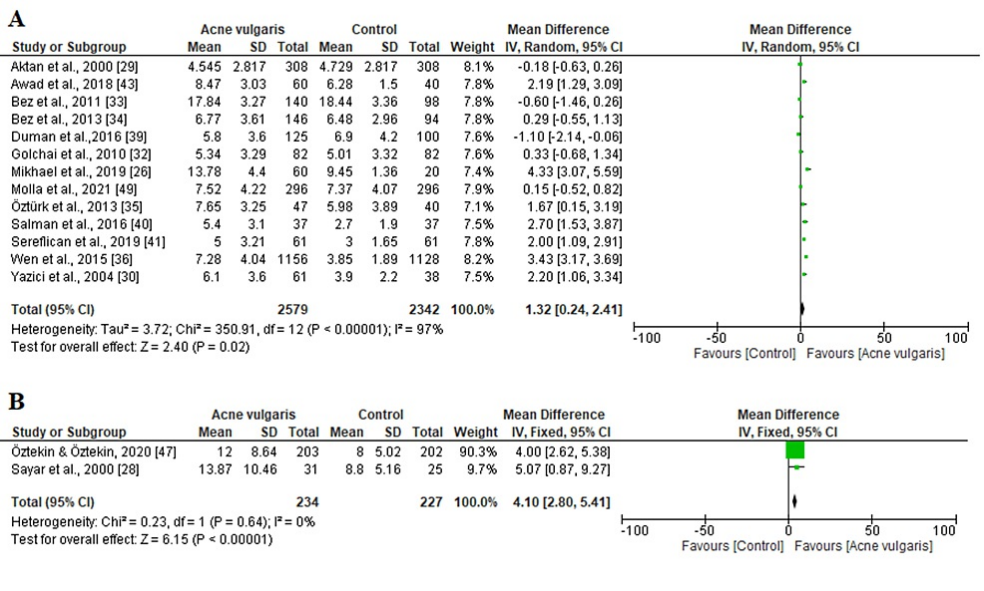
Forest plots demonstrating the pooled estimated mean difference of (A) HADS-D, (B) BDI scales between patients and the control group SD: standard deviation; IV: inverse variance, CI: confidence interval; HADS-D: Hospital Anxiety and Depression Scale-Depression Subscale; BDI: Beck Depression Inventory The solid vertical line represents a mean difference of 0 or no effect. Each square around the point effect represents the mean effect size for that study and reflects the relative weighting of the study to the overall effect size estimate. The larger the box, the greater the study's contribution to the overall estimate. The upper and lower limits of the line connected to the square represent the upper and lower 95% CI for the effect size.

Depression outcomes: Of the 23 included studies, 13 reported the HADS-D scale, with a total of 4921 participants. We used a random-effects design due to the high heterogeneity (chi^2^=350.91, P<0.00001, I^2^=97%, Figure 5A). The results indicated that there was a significant difference in terms of the HADS-D score between patients and controls (MD=1.32; 95% CI: 0.24 to 2.41; P=0.02). In contrast to HADS-D, only two studies reported the BDI scale with a low level of heterogeneity (chi^2^=0.23, P=0.64, I^2^=0%; Figure 5B). Similarly, BDI data showed that depression was significantly higher among patients than in the control group (MD=4.10; 95% CI: 2.80 to 5.41; P < 0.00001).

**Figure 6:**
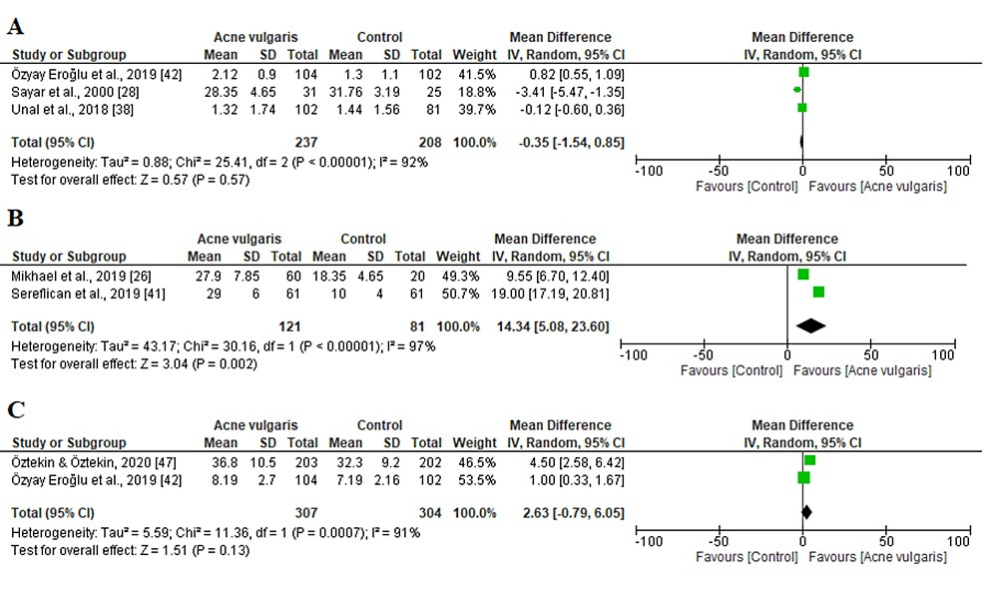
Forest plots showing the pooled estimated mean difference of the (A) RSS, (B) PSS, and (C) UCLA-LS scales between patients and the control group SD: standard deviation; IV: inverse variance; CI: confidence interval; RSS: Rosenberg Self-Esteem Scale; PSS: Perceived Stress Scale; UCLA-LS: University of California Los Angeles Loneliness Scale The solid vertical line represents a mean difference of 0 or no effect. Each square around the point effect represents the mean effect size for that study and reflects the relative weighting of the study to the overall effect size estimate. The larger the box, the greater the study's contribution to the overall estimate. The upper and lower limits of the line connected to the square represent the upper and lower 95% CI for the effect size.

Self-esteem, stress, and loneliness outcomes:* *Of the 23 included studies, three reported the RSS scale while only two investigated the PSS and UCLA-LS scales (Figure 6). The heterogeneity was high (P < 0.05, I^2^ > 80%); consequently, a random-effects design was adopted for all outcomes. We revealed that the difference between patients and controls in terms of self-esteem and loneliness outcomes was not statistically significant (P>0.05) (Figures 6A, 6C). However, the stress scale was significantly higher among patients than in the control group (MD=14.34; 95% CI: 5.08 to 23.60; P < 0.00001) (Figure 6B).

**Figure 7:**
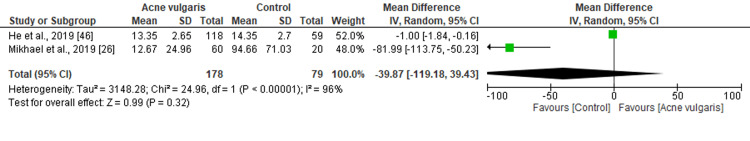
Forest plot showing the pooled estimated mean difference of BDNF between patients and the control group SD: standard deviation; IV: inverse variance; CI: confidence interval; BDNF: Brain‐Derived Neurotrophic Factor The solid vertical line represents a mean difference of 0 or no effect. Each square around the point effect represents the mean effect size for that study and reflects the relative weighting of the study to the overall effect size estimate. The larger the box, the greater the study's contribution to the overall estimate. The upper and lower limits of the line connected to the square represent the upper and lower 95% CI for the effect size.

BDNF outcomes: Two studies analyzed BDNF outcomes, with a total of 257 participants. The heterogeneity was high (chi^2^=24.96, P < 0.000001, I^2^=96%) so a random effect model was adopted (Figure 7). We noticed that the BDNF level was higher among the control group than the patients (MD=-39.87; 95% CI: -119.18 to 39.43). However, no significant difference was detected (P=0.32).

Subgroup and Sensitivity Analyses

We carried out subgroup and sensitivity analyses for anxiety and depression outcomes. The remaining outcomes were excluded given that the number of articles was limited. The mean difference in anxiety and depression among patients and the control group were different by the geographic origin of the work, the model of study, and the measurement tool adopted to assess outcomes. When the geographic origin of the work was adopted as a moderator, the mean difference in anxiety differed between studies. Indeed, the highest mean difference in anxiety in patients with acne vulgaris was detected in Egypt (MD=4.78) followed by China (MD=2.94). Furthermore, the mean difference in anxiety differed depending on the tool used for assessment. The mean difference in anxiety was high (MD=11.83, 95% CI=2.99-20.68) when it was measured by using LSAS, compared with ASI-3 (MD=4.13, 95% CI=2.51-5.75) and HADS-A (MD=1.86, 95% CI=0.96-2.76). The mean difference in anxiety significantly differed according to the study design adopted. Indeed, the mean difference in anxiety was higher in cross-sectional studies (MD=2.97, 95% CI=1.84-4.10) than in case-control studies (MD=1.53, 95% CI=0.43-2.62) (Table 3).

**Table 3:** Subgroup analyses for (A) anxiety and (B) depression outcomes HADS-A: Hospital Anxiety and Depression Scale-Anxiety Subscale; LSAS: Liebowitz Social Anxiety Scale; ASI-3: Anxiety Sensitivity Index; HADS-D: Hospital Anxiety and Depression Scale-Depression Subscale; BDI: Beck Depression Inventory

Subgroups	No. of studies	Mean difference	95% confidence interval	Heterogeneity		
				I^2^	Chi^2^	P
A- Anxiety						
Country studies conducted						
Turkey	9	1.50	0.38-2.63	91%	84.79	<0.001
Egypt	1	4.78	3.56-6.00	0	0	
Saudi Arabia	1	0.83	0.08-1.58	0	0	
China	1	2.94	2.68-3.20	0	0	
Iran	1	2.07	1.06-3.08	0	0	
Assessment tool used						
HADS-A	13	1.86	0.96-2.76	94%	188.50	<0.001
LSAS	3	11.83	2.99-20.68	88%	16.09	
ASI-3	2	4.13	2.51-5.75	8%	1.09	
Study design						
Case-control	10	1.53	0.43-2.62	95%	176.98	<0.001
Cross-sectional	4	2.97	1.84-4.10	68%	6.26	
B- Depression						
Country studies conducted						
Turkey	10	0.97	0.08-1.87	89%	73.18	<0.001
Egypt	1	4.33	3.07-5.59	0	0	
Saudi Arabia	1	0.15	-0.52-0.82	0	0	
China	1	3.43	3.17-3.69	0	0	
Iran	1	0.33	-0.68-1.34	0	0	
Assessment tool used						
HADS-D	12	1.32	0.24-2.41	97%	350.91	<0.001
BDI	2	4.10	2.80-5.41	0%	0.23	
Study design						
Case-control	11	1.22	-0.10-2.54	97%	339.43	<0.001
Cross-sectional	4	1.66	0.32-3.00	80%	10.19	

Similar results were revealed with depression scores. Indeed, the mean difference in depression among patients with acne vulgaris was much higher in Egypt (MD=4.33) than in China (MD=3.43). Moreover, the mean difference in depression differed depending on the tool used for assessment. The BDI scale revealed a higher mean difference (MD=4.10, 95% CI=2.80-5.41) than the HADS-D scale (MD=1.32, 95% CI=0.24-2.41). The mean difference in depression significantly differed according to the study design adopted. Indeed, the mean difference in depression was higher in cross-sectional studies (MD=1.66, 95% CI=0.32-3.00) than in case-control studies (MD=1.22, 95% CI=-0.10-2.54) (Table 3). Additionally, to further reveal the likely origin of heterogeneity among HADS-D and HADS-A outcomes, a leave-one-out sensitivity analysis was performed. We revealed that the outcomes did not differ markedly, which indicates that the meta-analysis had strong reliability. Indeed, the mean difference between HADS-A and HADS-D ranged from 1.64 (95% CI 0.67-2.54) to 1.99 (95% CI 0.96-2.94), and from 1.10 (95% CI 0.08-2.29) to 1.53 (95% CI 0.52-2.68), respectively (Table 4).

**Table 4:** Sensitivity analyses of mean difference in terms of (A) anxiety (HDAS-A) and (B) depression (HDAS-D) among patient and control group. HADS-A: Hospital Anxiety and Depression Scale-Anxiety Subscale; HADS-D: Hospital Anxiety and Depression Scale-Depression Subscale

Study excluded	Mean difference (95% CI)
HADS-A	
Aktan et al., 2000 [29]	1.80 (0.89-2.70)
Awad et al., 2018 [43]	1.74 (0.80-2.60)
Bez et al., 2011 [33]	1.95 (0.93-2.82)
Bez et al., 2013 [34]	1.92 (0.90-2.80)
Duman et al., 2016 [39]	1.65 (0.67-2.54)
Golchai et al., 2010 [32]	1.68 (0.69-2.58)
Mikhael et al., 2019 [26]	1.99 (0.96-2.94)
Molla et al., 2021 [49]	1.85 (0.92-2.76)
Ozturk et al., 2013 [35]	1.80 (0.89-2.70)
Salman et al., 2016 [40]	1.64 (0.67-2.54)
Sereflican et al., 2019 [41]	1.88 (0.93-2.78)
Wen et al., 2015 [36]	1.79 (0.83-2.63)
Yazici et al., 2004 [30]	1.90 (0.90-2.81)
HADS-D	
Aktan et al., 2000 [29]	1.32 (0.24-2.41)
Awad et al., 2018 [43]	1.30 (0.23-2.40)
Bez et al., 2011 [33]	1.25 (0.20-2.38)
Bez et al., 2013 [34]	1.11 (0.10-2.30)
Duman et al., 2016 [39]	1.40 (0.30-2.49)
Golchai et al., 2010 [32]	1.45 (0.38-2.53)
Mikhael et al., 2019 [26]	1.28 (0.14-2.34)
Molla et al., 2021 [49]	1.32 (0.24-2.41)
Ozturk et al., 2013 [35]	1.35 (0.28-2.45)
Salman et al., 2016 [40]	1.10 (0.08-2.29)
Sereflican et al., 2019 [41]	1.49 (0.44-2.62)
Wen et al., 2015 [36]	1.53 (0.52-2.68)
Yazici et al., 2004 [30]	1.18 (0.15-2.34)

Publication Bias

**Figure 8:**
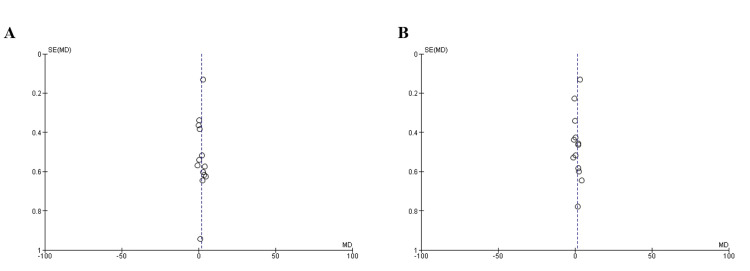
Funnel plots demonstrating no evidence of publication bias among the included articles in terms of (A) anxiety (HADS-A) and (B) depression (HADS-D) scores HADS-A: Hospital Anxiety and Depression Scale-Anxiety Subscale; HADS-D: Hospital Anxiety and Depression Scale-Depression Subscale

We demonstrated no proof of publication bias for HADS-A and HADS-D scores using Egger’s regression test (P=0.42, P=0.31, respectively). Moreover, a visual inspection of the funnel plot revealed a symmetrical funnel (Figure 8).

Discussion

Acne lesions represent a long-term skin condition, which leads to important psychiatric effects on the patients. Acne vulgaris notably affects the quality of life and is linked to psychosocial burden [36]. In this study, two measures analyzing the quality of life, AQOL and DLQI, revealed that the difference between men and women was not statistically significant.

The results of precedent studies on the association between QOL and gender seem to be highly conflicting [30,31,38]. Abdel-Hafez et al. reported that men suffering from acne vulgaris presented with QOL scores more deteriorated than women. Seeing that, in Egypt, most women are housewives, they are not as subject to social discomfort as men. On the other hand, Kellett and Gawkrodger demonstrated that women suffering from acne vulgaris experienced significantly lower life quality than men [50].

Furthermore, patients suffering from acne vulgaris demonstrated a higher score on the SCL 90-R subscale than those in the comparison group, but no significant difference was detected. This shows that psychiatric conditions are generally more frequent in patients with acne. Certainly, acne vulgaris leads to psychiatric disorders, including emotional distress and social anxiety, which may cause suicidal ideation [51].

Regarding anxiety, the HADS-A, LSAS, and ASI-3 scores were significantly higher among patients than among healthy participants. These results support the hypothesis that people with acne vulgaris have higher anxiety sensitivity compared to healthy participants. In this regard, Yazici et al. confirmed that acne vulgaris is associated with anxiety [30]. Similarly, diverse studies demonstrated a greater degree of social anxiety and behavior change (e.g. avoidance) in people presenting acne vulgaris compared with healthy participants [35,40,41,44]. It may be expected that people suffering from skin conditions would present more social anxiety. In addition, it was shown that women had a heightened degree of anxiety than men in the acne group, which proposes that the relationship between acne and anxiety may be linked to sex [52]. Contrarily, some researchers have reported conflicting results and have failed to address the association between acne vulgaris and social anxiety [29], suggesting that the psychosocial consequences are multifactorial.

In addition to anxiety, this meta-analysis detected a significantly higher level of depression in the patient group compared to the control group, as revealed by the HADS-D and BDI scales (P < 0.05). Similarly, many reports have revealed that psychosocial conditions, in particular, anxiety and depression, are more frequent among people presenting with acne vulgaris [52-54]. In addition, patients with a great degree of anxiety are most likely to have an increased degree of depression. Social loneliness, troubles at school or work, and love relationships, are the consequences of elevated social anxiety and can induce the appearance of depression in infected people. Zhang et al. found that patients with skin conditions were associated with a high incidence of anxiety and depression disorders, and suicidal ideations as compared with the control group [55]. Contrary to anxiety, Aktan et al. reported that depression among girls and boys with acne vulgaris was not significantly different [29]. Similarly, it was found that the difference in terms of depression outcomes between women and men in a group of patients suffering from mixed dermatological disorders, was not statistically significant [53].

Patients suffering from acne vulgaris presented a low level of self-esteem and body satisfaction not only because of their self-image but also because of the psychosocial judgments of others [55]. Considering that acne vulgaris is visible on the face and presents a specific level of social anxiety, it was suggested that severe as well as mild acne vulgaris may reduce the self-confidence and willingness of patients. Similarly, it was reported that the feeling of loneliness was higher among adolescents with acne [47]. Consequently, a high level of internet addiction was detected among them. The self-reported stress levels, measured by PSS score, were notably elevated in patients compared to healthy participants (MD=14.34; P < 0.00001). Similar results have been reported by Aslan Kayiran et al. and Zari and Alrahmani. Studies suggest that acne has a significant influence on emotional stress [13,56]. Similarly, there is an association between the stress scale and the severity of acne vulgaris. Indeed, acne exacerbates under stressful conditions. The pathogenesis of acne vulgaris is complex and multifactorial. One of the four main factors that play a role in the pathogenesis of acne vulgaris is an increase in sebum production. The increase in sebum production is one of the main factors that contribute to the pathogenesis of acne vulgaris. This factor can be triggered by the rise in corticotropin-releasing hormone (CRH) that is stimulated during stress. Moreover, stress also stimulates other neuropeptides that cause neurogenic inflammation and lead to the proliferation of the pilosebaceous glands [57].

Although this meta-analysis revealed the decrease of BDNF concentrations in the patient group in comparison with healthy participants, there is still a lack of evidence to conclude that it can be used as a specific indicator for evaluating the mental stress of patients with acne vulgaris seeing that no significant difference was detected between both groups. Similarly, Mikhael et al. showed statistically notable negative correlations between serum levels of BDNF and PSS scores, suggesting the role of BDNF as an important prognosis factor for the evaluation of stress in patients with acne vulgaris. Moreover, they revealed that the concentration of BDNF in patients with severe acne was notably lower compared to its concentration in patients with mild cases [26]. In the same way, He et al. showed that serum BDNF concentrations were negatively correlated with depression in patients suffering from acne vulgaris [46]. The association between BDNF and depression was highlighted at the molecular level. Certain reports revealed that Val66-Met polymorphism in the BDNF gene may be a significant genetic predisposition for depression [58,59].

Thus, further research based on standardized methodology and with a larger sample size is required to better understand the impact of psychiatric troubles among patients with acne vulgaris. 

## Conclusions

To the best of our knowledge, this work constitutes the first systematic review and meta-analysis to assess the psychiatric comorbidities, quality of life, and BDNF level in people suffering from acne vulgaris compared to the control group. To summarize, this meta-analysis reveals that acne vulgaris and psychological disorders (anxiety, depression, and stress) are significantly correlated. With reference to BDNF evaluation, although the score can be used as an indicator of mental stress, there is still a lack of evidence to conclude that it can be used as a specific indicator for evaluating the mental stress of acne vulgaris patients. Research studies with large sample sizes should be performed to confirm these results. Our findings emphasize the necessity of an interdisciplinary strategy between dermatologists and psychiatrists to provide better care and quality treatment for patients with acne vulgaris.
